# Measuring Alterations of Spontaneous EEG Neural Coupling in Alzheimer's Disease and Mild Cognitive Impairment by Means of Cross-Entropy Metrics

**DOI:** 10.3389/fninf.2018.00076

**Published:** 2018-10-30

**Authors:** Saúl J. Ruiz-Gómez, Carlos Gómez, Jesús Poza, Mario Martínez-Zarzuela, Miguel A. Tola-Arribas, Mónica Cano, Roberto Hornero

**Affiliations:** ^1^Biomedical Engineering Group, University of Valladolid, Valladolid, Spain; ^2^IMUVA, Mathematics Research Institute, University of Valladolid, Valladolid, Spain; ^3^INCYL, Neuroscience Institute of Castilla y León, University of Salamanca, Salamanca, Spain; ^4^Imaging and Telematics Group, University of Valladolid, Valladolid, Spain; ^5^Department of Neurology, Río Hortega University Hospital, Valladolid, Spain; ^6^Department of Clinical Neurophysiology, Río Hortega University Hospital, Valladolid, Spain

**Keywords:** Alzheimer's disease, mild cognitive impairment, electroencephalography (EEG), neural coupling, cross-entropy metrics

## Abstract

Alzheimer's Disease (AD) represents the most prevalent form of dementia and is considered a major health problem due to its high prevalence and its economic costs. An accurate characterization of the underlying neural dynamics in AD is crucial in order to adopt effective treatments. In this regard, mild cognitive impairment (MCI) is an important clinical entity, since it is a risk-state for developing dementia. In the present study, coupling patterns of 111 resting-state electroencephalography (EEG) recordings were analyzed. Specifically, we computed Cross-Approximate Entropy (*Cross-ApEn*) and Cross-Sample Entropy (*Cross-SampEn*) of 37 patients with dementia due to AD, 37 subjects with MCI, and 37 healthy control (HC) subjects. Our results showed that *Cross-SampEn* outperformed *Cross-ApEn*, revealing higher number of significant connections among the three groups (Kruskal-Wallis test, FDR-corrected *p*-values < 0.05). AD patients exhibited statistically significant lower similarity values at θ and β_1_ frequency bands compared to HC. MCI is also characterized by a global decrease of similarity in all bands, being only significant at β_1_. These differences shows that β band might play a significant role in the identification of early stages of AD. Our results suggest that *Cross-SampEn* could increase the insight into brain dynamics at different AD stages. Consequently, it may contribute to develop early AD biomarkers, potentially useful as diagnostic information.

## 1. Introduction

The human brain is an extremely complex network comprised of billions of interconnected neurons (Babiloni et al., 2016). Abnormal neural patterns at cellular coupling can provoke cognitive, behavioral, and functional alterations. Dementia due to Alzheimer's disease (AD) is the most common cause of neurodegenerative pathology, affecting up to 38% of people over 85 years (Alzheimer's Association, 2017). Neural activity in AD is progressively modified as a consequence of the neurodegenerative process and disturbances in information transmission and processing in the brain arise (Babiloni et al., 2016). Current interest in the field is focused on the detection of AD at its earliest possible stages. In this regard, mild cognitive impairment (MCI) appears as an important clinical entity, since it is considered as a prodromal stage of AD. Previous research have shown that MCI subjects progress to AD at a rate of approximately 15% per year (Davatzikos et al., 2011), whereas healthy controls develop dementia at a rate of 1–2% per year (Alzheimer's Association, 2017). Thus, MCI can be considered a prodromal form of AD. MCI subjects exhibit objective evidence of memory impairment greater than expected for their age and education level. Nonetheless, MCI does not necessarily interfere in their daily activities (Petersen, 2010).

Several techniques have been used to study neural dynamics in AD and MCI, such as positron emission tomography (PET), functional magnetic resonance imaging (fMRI), electroencephalography (EEG), and magnetoencephalography (MEG) (Ewers et al., 2011). In this study, the electrical brain activity was measured via EEG due to its high temporal resolution in contrast to PET and fMRI, which offer lower temporal resolution (Poza et al., 2014). Moreover, EEG is a non-invasive technique widely used in clinical settings to take advantage of its low cost compared to MEG. EEG measures the electrical activity of the brain generated by synchronized neurons (Poza et al., 2017). This information can help to further understand the relationship between neuronal dynamics and the alterations in brain function (Vecchio and Babiloni, 2011; Babiloni et al., 2016). Moreover, EEG has already shown its usefulness to characterize brain dynamics in AD and MCI (Koenig et al., 2005; Babiloni et al., 2009; Dauwels et al., 2010b).

In past decades, the abnormalities in AD and MCI neural activity were typically characterized using local activation analyses in individual sensors, by means of spectral and non-linear measures. Spectral analyses reflect a power increase in low frequency bands as the disease worsens, and a decrease in higher frequencies (Baker et al., 2008; Gasser et al., 2008; Ruiz-Gómez et al., 2018). Parameters derived from non-linear techniques have revealed that AD and MCI are characterized by a decrease in complexity and variability (Jeong, 2004; Dauwels et al., 2010a; McBride et al., 2014; Ruiz-Gómez et al., 2018). Particularly, different notions of entropy, such as Approximate Entropy (*ApEn*) and Sample Entropy (*SampEn*), have paid great attention in discriminating AD patients, MCI subjects, and cognitively healthy control (HC) subjects, mostly using binary approaches. In these studies, AD patients showed significant lower *ApEn* and *SampEn* values than MCI patients and HC subjects (Abásolo et al., 2005, 2006; Hornero et al., 2008; Gómez et al., 2009). These results support the well-known hypothesis that the EEG activity becomes more regular as the disease progresses.

However, spectral and non-linear parameters are no longer sufficient for a full characterization of brain dynamics (Stam and van Straaten, 2012). For this reason, increasing efforts have been made to gain further understanding of how the brain is organized as a functional network. Similarities between time series are traditionally quantified with linear methods, such as coherency and spectral estimations (Koenig et al., 2005; Babiloni et al., 2006; Moretti et al., 2008; Frantzidis et al., 2014b; Tóth et al., 2014). Nevertheless, these methods are not suitable for characterizing non-stationary signals. For this reason, most of them only found subtle alterations that depend on the particular coupling parameter. Since different entropy-based measures, such as *ApEn* and *SampEn*, are very well-suited to analyze short and noisy one-dimensional time series (Pincus, 1991), the multidimensional versions of these methods are a good option for the analysis of multiple signals recorded from many electrodes, like EEG. Cross-Approximate Entropy (*Cross-ApEn*) and Cross-Sample Entropy (*Cross-SampEn*) algorithms can be applied to two signals, to quantify the statistical similarity between them (Pincus, 2001). It is neccessary to address that *Cross-ApEn* has some limitations: it is not consistent for every condition and is not always defined. *Cross-SampEn* was proposed to overcome these drawbacks, remaining relatively consistent for all conditions and being always defined (Richman and Moorman, 2000). Finally, there is other important difference between these two measures: whereas *Cross-ApEn* analysis exhibits direction dependence (i.e., it is an asymmetric method), *Cross-SampEn* is a direction independent measure. Usually, higher values of cross-entropy metrics indicate less similarity between signals, and they are associated with weaker coupling (Hudetz et al., 2003). Only a few studies have applied *Cross-ApEn* and *Cross-SampEn* to biological systems (Pincus and Singer, 1996; Licinio et al., 1998; Martínez-Zarzuela et al., 2013). To the best of our knowledge, only our preliminary study has analyzed spontaneous EEG activity in AD by means of *Cross-SampEn* and graph theory parameters (Gómez et al., 2016).

In this study, we hypothesized that the coupling patterns between different functional brain regions are disrupted in dementia, even at prodromal stages. These alterations involved in cognitive decline affect the EEG activity and could be characterized by means of cross-entropy metrics. Accordingly, in the current research we attempt to address the following questions: (i) can *Cross-SampEn* overcome the *Cross-ApEn* technical drawbacks and provide additional information about spontaneous EEG activity? (ii) which of these measures yield a better characterization of the abnormal coupling patterns in AD and MCI? and (iii) can these metrics be useful to discriminate AD and MCI patients from HC subjects?

## 2. Materials

### 2.1. Participants

In this study, we recruited a total of 111 subjects: 37 AD patients (12 males and 25 females), 37 MCI patients (16 males and 21 females), and 37 age-matched HC subjects (12 males and 25 females). Patients with dementia due to AD and MCI were diagnosed following the criteria of the National Institute on Aging and Alzheimer's Association (NIA-AA). HC volunteers had no pathological background and underwent a medical examination and cognitive assessment in order to discard any symptoms of neurological disorder (Albert et al., 2011). Exclusion criteria were the same used in our previous studies (Poza et al., 2017; Ruiz-Gómez et al., 2018): (1) presence or history of another neurological or psychiatric disease, different from MCI or dementia due to AD; (2) atypical course or uncommon clinical presentations according to the NIA-AA criteria; (3) advanced dementia (Clinical Dementia Rating = 3); (4) institutionalized patients; (5) medication that could affect EEG activity; and (6) lack of cooperation during EEG acquisition. This study was carried out in accordance with the recommendations of the Code of Ethics of the World Medical Association with written informed consent from all subjects. All subjects and caregivers gave written informed consent in accordance with the Declaration of Helsinki. The protocol was approved by The Ethics Committee at the Río Hortega University Hospital (Valladolid, Spain). Relevant socio-demographic and clinical characteristics are specified in Tables 1, 2 for training and test sets, respectively.

**Table 1:** Socio-demographic and clinical data for each group in the training set.

	**HC**	**MCI**	**AD**
Number of subjects	20	20	20
Number of trials	912	937	917
Age (years) (median[IQR])	75.6[74.1, 77.6]	77.9[67.9, 79.8]	80.7[74.7, 78.9]
Gender (Male:Female)	8:12	8:12	5:15
MMSE^a^ (median[IQR])	29[28, 30]	27.5[26.5, 29]	21[18.5, 22.5]
B-ADL^b^ (median[IQR])	1.1[1.0, 1.2]	2.9[2.4, 3.3]	5.8[5.1, 7.2]
Education level (A:B)^*c*^	5:15	11:9	8:12

a *MMSE, Mini Mental State Examination*;

b *B-ADL, Bayer-Activities of Daily Living*;

c *A, Primary education or below; B, Secondary education or above*.

**Table 2:** Socio-demographic and clinical data for each group in the test set.

	**HC**	**MCI**	**AD**
Number of subjects	17	17	17
Number of trials	752	847	757
Age (years) (median[IQR])	76.4[73.6, 78.9]	75.3[69.8, 82.0]	82.4[77.7, 83.9]
Gender (Male:Female)	4:13	8:9	7:10
MMSE^a^ (median[IQR])	29[28, 30]	27[27, 28]	22[20, 24]
B-ADL^b^ (median[IQR])	1.2[1.0, 1.3]	2.8[2.3, 2.5]	6.4[5.0, 7.3]
Education level (A:B)^*c*^	5:12	12:5	10:7

a *MMSE, Mini Mental State Examination*;

b *B-ADL, Bayer-Activities of Daily Living*;

c *A, Primary education or below; B, Secondary education or above*.

### 2.2. EEG recording

Resting-state EEG activity was acquired using a 19-channel EEG system (XLTEK®, Natus Medical) at a sampling frequency of 200 Hz. The electrodes were located at the positions Fp1, Fp2, Fz, F3, F4, F7, F8, Cz, C3, C4, T3, T4, T5, T6, Pz, P3, P4, O1, and O2 according to the international 10–20 system. Common average referencing (CAR) was chosen as the reference technique for EEG recoding because previous studies found that CAR outperforms standard types of electrical referencing (Ludwig et al., 2009). Subjects were asked to remain awake with closed eyes during EEG acquisition. For each five-minute EEG recording, the following pre-processing procedure was applied: (i) digital filtering using a Hamming window bandpass finite impulse response (FIR) filter between 0.4 and 98 Hz and a notch filter to remove the power line frequency interference (50 Hz); (ii) independent component analysis (ICA) to minimize the presence of oculographic, cardiographic, and myographic artifacts; (iii) digital filtering using a Hamming window bandpass FIR filter in the band of interest (1–70 Hz); and (iv) selection of 5-s artifact-free epochs by visual inspection.

In our previous study (Ruiz-Gómez et al., 2018), we randomly divided the EEG database into training and test sets. The training set was composed of 60 subjects (20 of each group), while the remaining 51 subjects were assigned to the test set (17 of each group). In addition, for every comparison between groups no statistically significant differences were found in age and gender (*p*-value > 0.05, Kruskal-Wallis test and chi-squared test, respectively). In order to compare our results with the previous ones, training and test sets remain unchanged (Ruiz-Gómez et al., 2018).

## 3. Methods

The followed methodology is explained below:

1. Training set:
  a. First, after data collection and pre-processing, *Cross-ApEn* and *Cross-SampEn* were computed in the following frequency bands: delta (δ, 1–4 Hz), theta (θ, 4–8 Hz), alpha (α, 8–13 Hz), beta-1 (β_1_, 13–19 Hz), beta-2 (β_2_, 19–30 Hz), and gamma (γ, 30–70 Hz). Also, both measures were computed for different combinations of their configuration parameters, the run length *m* and the tolerance window *r*. The optimal values for *m* and *r* were obtained by evaluating the ranges suggested by Pincus (Pincus, 2001): *m*∈[1, 2] and *r*∈[0.10, 0.15, 0.20, 0.25]. The result of computing each measure for all pair-wise combinations of channels was an *M*×*M* matrix (*M* = 19), where each entry *M*_*i, j*_ contains the *Cross-ApEn* or *Cross-SampEn* between the channels *i* and *j*.
  b. Then, we selected the parameters combination for which the corresponding *Cross-ApEn* or *Cross-SampEn* values showed the highest number of significant connections among the three groups using false discovery rate (FDR) (Benjamini and Hochberg, 1995) (FDR-corrected *p*-values < 0.05, Kruskal-Wallis test).
  c. After determining the measure and *m* and *r* values that provides a better discrimination among the three groups, fast correlation-based filter (FCBF) (Yu and Liu, 2004) was applied to select two optimal sets of connections for discriminating HC vs. MCI and HC vs. AD, respectively.
  d. Afterwards, quadratic discriminant analysis (QDA), support vector machines (SVM), and decision trees (DT) models were trained with these optimal sets of features using training data.
2. Test set:
  e. For the chosen metric in step (b), coupling patterns were obtained for the subjects comprised in the test set. Statistical differences were evaluated between groups for HC vs. MCI and HC vs. AD comparisons.
  Finally, the binary discrimination ability of the optimal sets of features obtained were evaluated by means of QDA, SVM, and DT approaches trained in step (d) using test data.

### 3.1. Cross-approximate entropy

*Cross-ApEn* quantifies the statistical dissimilarity between two paired signals (Pincus, 2000). The *Cross-ApEn* algorithm is quite similar to *ApEn*, but it is applied to two time series rather than an individual signal. Thus, *Cross-ApEn* affords a coupling metric from which you can directly determine the changes in interconnected networks (Pincus, 2000). The procedure for *Cross-ApEn* estimation requires two time series, *u* and *v*, of *N* samples. It is also necessary to determine the value of the run length *m* and the tolerance window *r*. Conceptually, *Cross-ApEn* quantifies the asynchrony between two time series by determining the frequency in which *m*-length patterns in *v* are similar to reference *m*-length patterns in *u* within a tolerance *r*.

Given the aforementioned time series *u* = [*u*(1), *u*(2), …, *u*(*N*)] and *v* = [*v*(1), *v*(2), …, *v*(*N*)], the algorithm to calculate *Cross-ApEn* is described as follows (Pincus, 2000):

1. Normalize *u* and *v* into u^*^ and v^*^, by subtracting the mean of each time series and dividing by its standard deviation.
2. Form the sequences of *m* consecutive u^*^ and v^*^ values starting with the *i*th and *j*th point, respectively.
  (1) $$\begin{array}{r} x\left(i\right)=\left[u^{*}\left(i\right),u^{*}\left(i+1\right),\ldots ,u^{*}\left(i+m-1\right)\right] \end{array}$$
  (2) $$\begin{array}{r} y\left(j\right)=\left[v^{*}\left(j\right),v^{*}\left(j+1\right),\ldots ,v^{*}\left(j+m-1\right)\right] \end{array}$$
3. Compute the distance between *x*(*i*) and *y*(*j*), *d*[*x*(*i*), *y*(*j*)], defined as the maximum absolute difference of their scalar components:
  (3) $$\begin{array}{r} d\left[x\left(i\right),y\left(j\right)\right]=\underset{k=0,1,\ldots ,m-1}{\max}|u^{*}\left(i+k\right)-v^{*}\left(j+k\right)| \end{array}$$
4. For each *x*_*m*_(*i*), find the number of *j* so that *d*[*x*_*m*_(*i*), *x*_*m*_(*j*)] is smaller or equal to *r*, denoted as $N_{i}^{m}\left(r\right)$. Then, for *i* = 1, 2, …, *N* − *m* + 1, set:
  (4) $$\begin{array}{r} C_{i}^{m}\left(r\right)\left(v||u\right)=\frac{N_{i}^{m}\left(r\right)}{N-m+1} \end{array}$$
5. Obtain ϕ^*m*^(*r*), averaging the natural logarithm of $C_{i}^{m}\left(r\right)$ over *i*:
  (5) $$\begin{array}{r} \phi^{m}\left(r\right)\left(v||u\right)=\frac{1}{N-m+1}\overset{N-m+1}{\underset{i=1}{\sum }}\ln\;C_{i}^{m}\left(r\right)\left(v||u\right) \end{array}$$
6. Similarly, obtain *C*^*m*+1^(*r*) and then compute ϕ^*m*+1^(*r*) following similar steps:
  (6) $$\begin{array}{r} C_{i}^{m+1}\left(r\right)\left(v||u\right)=\frac{N_{i}^{m+1}\left(r\right)}{N-m+1} \end{array}$$
  (7) $$\begin{array}{r} \phi^{m+1}\left(r\right)\left(v||u\right)=\frac{1}{N-m+1}\overset{N-m+1}{\underset{i=1}{\sum }}\ln\;C_{i}^{m+1}\left(r\right)\left(v||u\right) \end{array}$$
7. Finally, *Cross-ApEn* is defined as:
  (8) $$\begin{array}{r} \text{Cross-ApEn}\left(r,m,N\right)\left(v||u\right)=\phi^{m}\left(r\right)\left(v||u\right)-\phi^{m+1}\left(r\right)\left(v||u\right) \end{array}$$

The absence of similar patterns between *u* and *v* may lead to non-defined values of *Cross-ApEn*. Thus, two correction strategies have been proposed to assign non-zero values in the absence of matches: *bias* 0 and *bias max* (Richman and Moorman, 2000). In this study, the *bias max* correction strategy has been applied (Martínez-Zarzuela et al., 2013). This strategy assigns the values $C_{i}^{m}\left(r\right)=C_{i}^{m+1}\left(r\right)$ and $C_{i}^{m+1}\left(r\right)={\left(N-m+1\right)}^{-1}$ if originally $C_{i}^{m}\left(r\right)=0$ and $C_{i}^{m+1}\left(r\right)=0$, respectively.

### 3.2. Cross-sample entropy

*Cross-SampEn* was proposed by Richman and Moorman to overcome the drawbacks of *Cross-ApEn* (Richman and Moorman, 2000). *Cross-SampEn* is always defined and remains relatively consistent for conditions where *Cross-ApEn* does not. As *Cross-ApEn, Cross-SampEn* allows assessing the degree of dissimilarity between two time series. To compute *Cross-SampEn* is also necessary to specify the values of the run length *m* and the tolerance window *r*. Thus, the algorithm to compute *Cross-SampEn* between the previously described time series, *u* and *v*, is the following (Richman and Moorman, 2000):

1. Normalize *u* and *v* into u^*^ and v^*^, by subtracting the mean of each time series and dividing by its standard deviation.
2. Form the sequences of *m* consecutive u^*^ and v^*^ values starting with the *i*th and *j*th point, respectively.
  (9) $$\begin{array}{r} x\left(i\right)=\left[u^{*}\left(i\right),u^{*}\left(i+1\right),\ldots ,u^{*}\left(i+m-1\right)\right] \end{array}$$
  (10) $$\begin{array}{r} y\left(j\right)=\left[v^{*}\left(j\right),v^{*}\left(j+1\right),\ldots ,v^{*}\left(j+m-1\right)\right] \end{array}$$
3. Compute the distance between *x*(*i*) and *y*(*j*), *d*[*x*(*i*), *y*(*j*)], defined as the maximum absolute difference of their scalar components:
  (11) $$\begin{array}{r} d\left[x\left(i\right),y\left(j\right)\right]=\underset{k=0,1,\ldots ,m-1}{\max}|u^{*}\left(i+k\right)-v^{*}\left(j+k\right)| \end{array}$$
4. For each *x*_*m*_(*i*), find the number of *j* so that *d*[*x*_*m*_(*i*), *x*_*m*_(*j*)] is smaller or equal to *r* with *i*≠*j*, denoted as $b_{i}^{m}\left(r\right)$. Then, for *i* = 1, 2, …, *N*−*m*, set:
  (12) $$\begin{array}{r} B_{i}^{m}\left(r\right)\left(v||u\right)=\frac{b_{i}^{m}\left(r\right)}{N-m} \end{array}$$
5. Define *B*^*m*^(*r*)(*v*||*u*) as:
  (13) $$\begin{array}{r} B^{m}\left(r\right)\left(v||u\right)=\frac{1}{N-m}\overset{N-m}{\underset{i=1}{\sum }}\ln\;B_{i}^{m}\left(r\right)\left(v||u\right) \end{array}$$
6. Similarly, define *A*^*m*^(*r*)(*v*||*u*) as 1/(*N* − *m*) times the number of *j* (*j* = 1, 2, …, *N* − *m* + 1), such the distance between *x*_*m*+1_(*i*) and *y*_*m*+1_(*i*) is less or equal to *r*. Then, calculate:
  (14) $$\begin{array}{r} A^{m}\left(r\right)\left(v||u\right)=\frac{1}{N-m}\overset{N-m}{\underset{i=1}{\sum }}\ln\;A_{i}^{m}\left(r\right)\left(v||u\right) \end{array}$$
7. Finally, *Cross-SampEn* is defined as:
  (15) $$\begin{array}{r} \text{Cross-SampEn}\left(r,m,N\right)\left(v||u\right)=-\ln\;\left[\frac{A^{m}\left(r\right)\left(v||u\right)}{B^{m}\left(r\right)\left(v||u\right)}\right] \end{array}$$

### 3.3. Statistical analysis

Firstly, a descriptive analysis was carried out to study the distribution of the coupling results. Kolmogorov–Smirnov and Shapiro–Wilks tests were used to evaluate the normality of the data, whereas Levene test was employed to assess the homogeneity of variances. As *Cross-ApEn* and *Cross-SampEn* results did not meet the parametric test assumptions, a non-parametric test was used. Statistical differences among three groups were evaluated by Kruskal–Wallis test, whereas statistical differences between HC and MCI subjects and between HC and AD subjects were evaluated with Mann-Whitney *U*-test. In order to correct for multiple comparisons, FDR controlling procedure was used (Benjamini and Hochberg, 1995).

Signal processing was carried out using Matlab (version R2017a, Mathworks, Natick, MA), whereas statistical analyses were computed using SPSS Statistics (version 20, IBM Corp, Armonk, NY).

### 3.4. Classification analysis

The binary classification performance of the optimum sets of features for each comparison (HC vs. MCI and HC vs. AD) was assessed by means of QDA, SVM, and DT. These techniques are widely employed for data classification from EEG recordings (Spyrou et al., 2016; Chriskos et al., 2018; Ruiz-Gómez et al., 2018). In order to compare our results with those of our previous study (Ruiz-Gómez et al., 2018), the performances of the models were described by the same statistical measures: accuracy (*Acc*), sensitivity (*Se*), specificity (*Sp*), positive predictive value (*PPV*), and negative predictive value (*NPV*).

#### 3.4.1. Quadratic discriminant analysis (QDA)

QDA is commonly used due to its advantages over linear discriminant analysis (LDA). While LDA assumes both data normality (Gaussian or normal distribution) and homoscedasticity (equal variances) to model each class-conditional density function for an input feature, QDA does not presume homoscedasticity (Bishop, 2007). Then, in order to predict the classes of new data, the QDA models find the class with the smallest misclassification by establishing a quadratic decision boundary between classes in the feature space, instead the linear decision threshold of LDA (Bishop, 2007).

#### 3.4.2. Support vector machines (SVM)

SVM is a binary classifier that searches for the optimal hyperplane boundary, built in a transformed high-dimensional space to maximize separation. The weight vector *w* is obtained by solving an optimization problem based on Lagrange multipliers η^*n*^, expressed as follows (Vapnik, 1999):

(16) $$\begin{array}{r} \text{w}=\underset{n\in S}{\sum }\eta^{n}t^{n}\varphi \left(x^{n}\right), \end{array}$$

where *t*^*n*^ is the target or desired output and φ(·) maps training vectors into the higher dimensional space. The output of the SVM classifier is expressed in terms of these support vectors as follows (Vapnik, 1999):

(17) $$\begin{array}{r} \text{y}=\underset{n\in S}{\sum }\eta^{n}t^{n}K\left(x^{n},x\right)+w_{0}, \end{array}$$

where *S* is a subset of the indices {1, …, *N*} corresponding to the support vector and *K*(·, ·) represents the inner product kernel function in the transformed space. In the present study, a polynomial kernel is used. This kernel represents the similarity of vectors in a feature space over polynomials of the original variables, allowing learning of non-linear models (Goldberg and Elhadad, 2008).

#### 3.4.3. Decision trees (DT)

DT models predict responses to data and can be viewed as a combination of models in which only one model is responsible for making predictions at any given point in input space. The input space is partitioned into cuboid regions, whose edges are aligned with the axes. For any new input *x*, the region it falls into is determined by starting at the top of the tree (root node) and following a path down to a specific leaf node according to the decision criteria at each node (Breiman et al., 1984).

## 4. Results

According to the proposed methodology, we obtained a *Cross-ApEn* and a *Cross-SampEn* coupling matrix value per subject and frequency band. These matrices were obtained by averaging results from all artifact-free trials from 5-min recordings of each subject.

### 4.1. Training set

In order to chose the values for *m* and *r* to compute *Cross-ApEn* and *Cross-SampEn*, only the training set was used. They were obtained by evaluating all the combinations for *m*∈[1, 2] and *r*∈[0.10, 0.15, 0.20, 0.25]. Table 3 shows the total number of significant connections among the three groups (FDR-corrected *p*-values < 0.05, Kruskal-Wallis test) as the sum for all frequency bands for each parameters combination. The results of Table 3 show that *Cross-SampEn* with *m* = 1 and *r* = 0.2 exhibits the higher number of significant connections. Therefore, we chose that configuration for further analyses.

**Table 3:** Total number of significant connections among the three groups (FDR-corrected *p*-values < 0.05, Kruskal-Wallis test) for each parameter combination in the training set.

	***Cross-ApEn***		***Cross-SampEn***	
	***m* = 1**	***m* = 2**	***m* = 1**	***m* = 2**
*r* = 0.10	182	0	468	0
*r* = 0.15	486	213	508	12
*r* = 0.20	403	228	514	48
*r* = 0.25	349	255	502	196

After determining the optimal metric, FCBF was applied to derive the two optimal sets for binary classifications tasks (HC vs. MCI and HC vs. AD). For this purpose, each connection between two electrodes in every frequency band was interpreted as a feature. Table 4 shows the FCBF-selected features that formed optimal sets for HC vs. MCI and HC vs. AD classification tasks. These sets of features were used to train the QDA, SVM, and DT models. For each classifier, two models were trained: one with the aim of classifying MCI and HC subjects, and the other one for AD patients vs. HC subjects comparison.

**Table 4:** Optimal FCBF sets of features for HC vs. MCI and HC vs. AD comparisons.

**Comparison**	**Selected features**			
*HC* vs. *MCI*	Fz-T4 (δ)	Cz-C3 (θ)	C4-Fp2 (α)	P3-Fz (β_1_)
*HC* vs. *AD*	F7-Fp2 (δ)	Fp1-C3 (θ)	T3-T5 (θ)	C3-Pz (γ)

### 4.2. Test set

**Figure 1:**
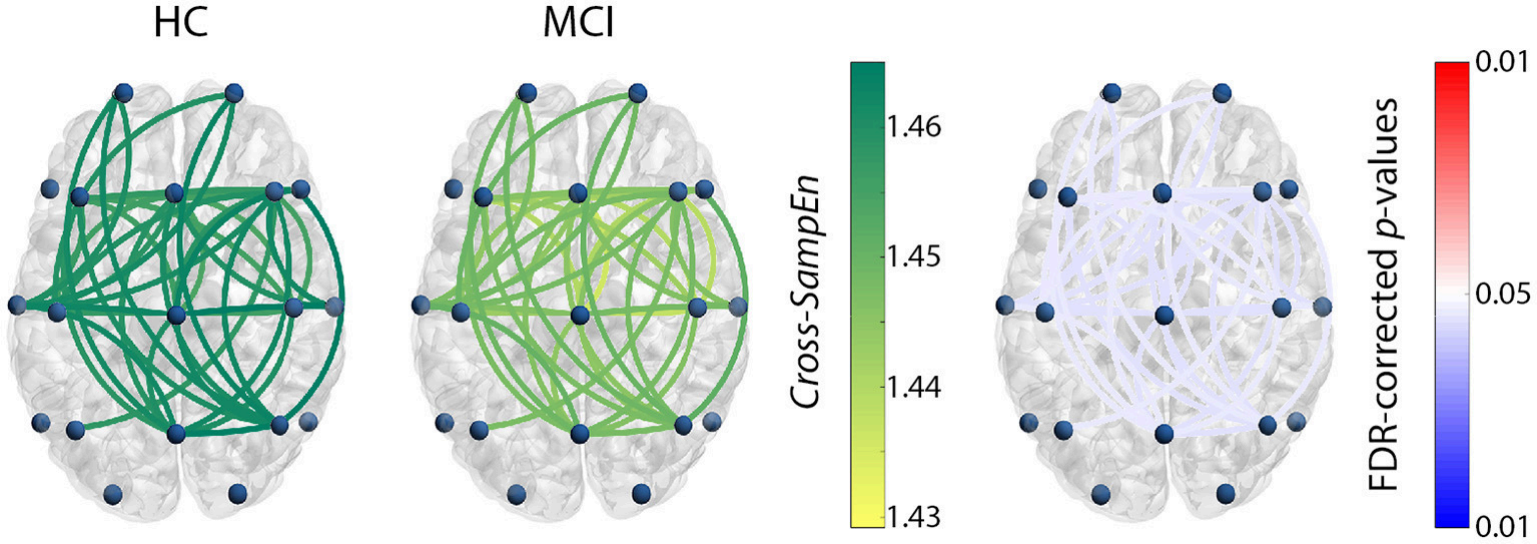
*Cross-SampEn* results for HC vs. MCI comparison at β_1_ band. Left and central columns depict *Cross-SampEn* values for controls and MCI patients, respectively. Right column displays statistical results, where connections were only displayed when statistically significant differences were obtained (FDR-corrected *p*-values < 0.05, Mann-Whitney *U*-test). Red color tones indicate significant *Cross-SampEn* increases in MCI compared with controls, whereas blue color tones denote significant decreases.

**Figure 2:**
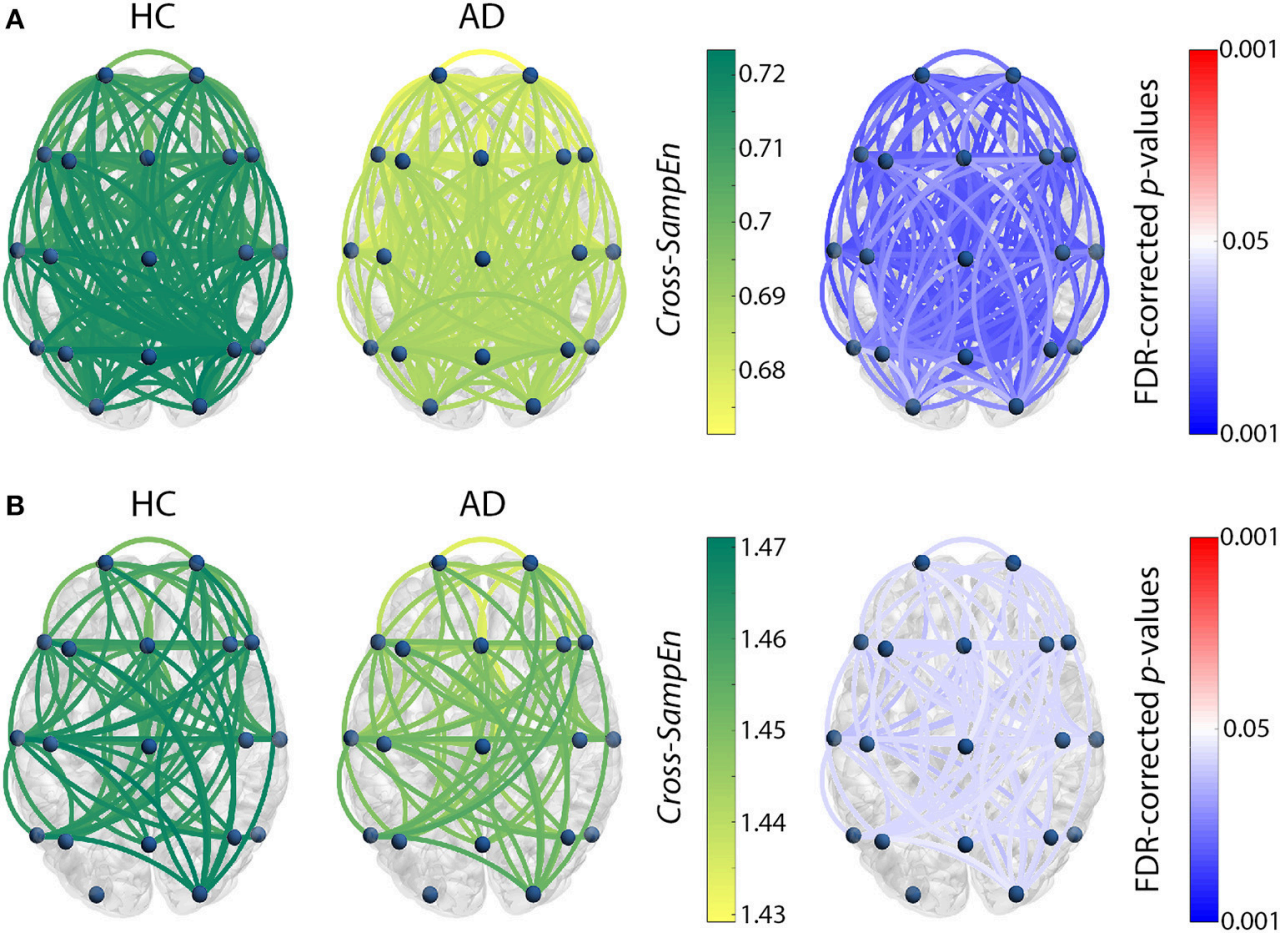
*Cross-SampEn* results for HC vs. AD comparison **(A)** at θ band, and **(B)** at β_1_ band. Left and central columns depict *Cross-SampEn* values for controls and AD patients, respectively. Right column displays statistical results, where connections were only displayed when statistically significant differences were obtained (FDR-corrected *p*-values < 0.05, Mann-Whitney *U*-test). Red color tones indicate significant *Cross-SampEn* increases in AD compared with controls, whereas blue color tones denote significant decreases.

For the previously chosen measure, *Cross-SampEn* (*m* = 1, *r* = 0.2), coupling patterns were obtained for the subjects comprised in the test set. Only those frequency bands that showed statistically significant connections are presented in Figure 1 (HC vs. MCI) and Figure 2 (HC vs. AD). In these figures, the left column shows *Cross-SampEn* values for the healthy group, while the outcomes for patients groups are presented in the center column. Finally, statistically significant connections (FDR-corrected *p*-values < 0.05, Mann-Whitney *U*-test) between groups are displayed in the right column using the following color-code: red color tones indicate significant *Cross-SampEn* increases in AD or MCI patients compared to controls, whereas blue color tones denote significant decreases. Note that for all columns, only statistically significant differences are displayed.

Our *Cross-SampEn* results showed that EEG activity in MCI patients is characterized by an overall similarity decrease in β_1_ band, as shown in Figure 1. Additionally, AD patients present significant higher *Cross-SampEn* values in θ and β_1_ frequency bands, as displayed in Figures 2A,B, respectively.

In order to evaluate the diagnostic ability of the previously trained models (QDA, SVM, and DT), only the test set was used. In the case of the HC vs. MCI classification task, higher diagnostic results were found with SVM and DT (*Acc* = 73.53%, *Se* = 58.82%, and *Sp* = 88.24%), compared with QDA (*Acc* = 67.65%, *Se* = 52.95, and *Sp* = 82.35). Also SVM and DT reached higher diagnostic performance for the HC vs. AD comparison (*Acc* = 82.35%, *Se* = 70.59%, and *Sp* = 94.12%) compared with QDA (*Acc* = 76.47%, *Se* = 76.47%, and *Sp* = 76.47%). Furthermore, both SVM and DT models present a good diagnostic capability for discriminating when a subject does not suffer AD (*Sp* = 94.12% and *NPV* = 76.19%) and MCI (*Sp* = 88.24% and *NPV* = 68.18%).

## 5. Discussion

The aim of this study was to assess the performance of *Cross-ApEn* and *Cross-SampEn* in order to find abnormal coupling patterns in the early stages of dementia. For that purpose, three main objectives based on the research questions were set.

### 5.1. Cross-ApEn vs. Cross-SampEn

The first research question was focused on determining whether *Cross-SampEn* can provide additional information about spontaneous EEG activity in AD and MCI compared to *Cross-ApEn*.

Only a few studies have applied these metrics to biological systems. For instance, *Cross-ApEn* has been applied to analyze secretory patterns of luteinizing hormone and testosterone in young and aged healthy men (Pincus and Singer, 1996), concentrations of circulating leptin, luteinizing hormone, and estradiol in healthy women (Licinio et al., 1998), blood oxygen saturation and heart rate signals from nocturnal oximetry (Álvarez et al., 2009), and bihemispheric EEGs from rats (Hudetz et al., 2003). On the other hand, Pritchard et al. (2014) applied *Cross-SampEn* to resting-state fMRI data with the aim of establish functional connectivity between different brain areas. To the best of our knowledge, only Xie et al. (2010) have compared the performance of *Cross-ApEn* and *Cross-SampEn* in order to prove the theoretical advantages of the last one. Firstly, they compared both measures quantitatively using five different coupled systems. Then, they applied both measures to a real-life problem in which they analyzed the synchronization patterns of left inter-hemisphere rats' EEG signals. Both analyses showed that *Cross-SampEn* could be more conveniently applied to different dynamical neural systems contaminated by noise.

Our results using the training set revealed a high number of significant connections among the three groups for all *Cross-SampEn* combinations with *m* = 1, as shown in Table 3. In our particular case, *Cross-SampEn* showed statistical differences among the three groups that *Cross-ApEn* could not detect. Therefore, taking into account that *Cross-SampEn* shows technical advantages over *Cross-ApEn* and our results, we could say that *Cross-SampEn* is more adequate to characterize the neural coupling patters in MCI and AD. Additionally, both metrics show better performances for *m* = 1 compared to *m* = 2. This could be due to the fast nature of EEG fluctuations which could be easier to detect with low values of *m*. Too large *m* values are inappropriate for detecting the dynamical changes in EEG recordings (Li et al., 2013). Finally, in order to avoid possible biases, it is important to optimize these configuration parameters for each particular database using a hold-out approach. That is, splitting the original dataset into a training set used to optimize these parameters and a test set used to measure the generalization performance of the metrics, as we have done in the current study.

### 5.2. Abnormal coupling patterns in MCI and AD

After comparing the usefulness of both measures and determining the optimal configuration, characterization of MCI and AD was assessed. Our *Cross-SampEn* (*m* = 1, *r* = 0.2) results revealed that MCI and AD groups are characterized by a global decrease of similarity in all frequency bands. However, significant differences were only found in β_1_ band for MCI and in θ and β_1_ bands for AD.

Previous EEG studies have shown evidences of coupling loss in AD through different connectivity measures. In line with our results, Besthorn et al. (1994) found a coherence decrease in AD, specifically in θ, α, and β bands. Synchronization likelihood (*SL*) also showed lower values for AD patients in all frequency bands, but they were statically significant only in the 14–18 and 18–22 Hz bands (corresponding with our β_1_ band, approximately) (Stam et al., 2003). Koenig et al. (2005) analyzed different databases using global field synchrony (*GFS*), finding significant differences in α and β bands. Furthermore, Jeong et al. (2001) reported lower values of cross-mutual information in AD subjects than in controls, confirming the disconnection syndrome in AD. To the best of our knowledge, only our previous study addressed the characterization AD by means of *Cross-SampEn* (Gómez et al., 2009), but the whole frequency range (1–40 Hz) was analyzed, instead of dividing it into the classical EEG bands. Our results in this preliminary study suggested that dementia due to AD is characterized by a lower degree of similarity among channels. The current research do not follow the same trend, since AD patients present widespread significant higher *Cross-SampEn* values in θ and β_1_ bands, with long-range connections being more common, since it is one of the main characteristics of pathological aging (Frantzidis et al., 2014a). Several authors have suggested that the observed abnormal coupling patterns may be due to the loss of acetylcholine, long-distance association fibers, or gray matter volume (Cook and Leuchter, 1996; Francis et al., 1999; Kikuchi et al., 2000; Karas et al., 2004; Cho et al., 2008). Acetylcholine is a major excitatory modulator of cortical synaptic function and the effect of blocking the cholinergic system is the reduction of resting EEG coupling (Francis et al., 1999; Kikuchi et al., 2000). Additionally, the loss of long distance association fibers produce interhemispheric corticocortical disconnection that could contribute to cognitive impairment (Cook and Leuchter, 1996; Cho et al., 2008). Finally, global and regional gray matter density loss in AD patients indicate an ongoing atrophic process in their brains, that could produce the disconnection between different brain areas (Karas et al., 2004). Other authors suggested that the neuron loss may cause the coupling decrease (Moretti et al., 2011). Nevertheless, if the loss of EEG coupling in AD would simply be caused by a loss of neurons, it would be difficult to understand why all frequencies are not equally affected (Stam et al., 2003).

Despite the fact that MCI studies are less common, a few ones have reported that this pathology is also associated with less connected brain networks. Connectivity decrease in MCI was revealed also by lower *SL* values in δ and α bands (Babiloni et al., 2006). This trend of lower *SL* values for MCI is also present in β band from MEG data (Gómez et al., 2009). Koenig et al. (2005) showed intermediate *GFS* values between AD and HC for MCI subjects in α and β frequency bands. The aforementioned studies provide evidences for considering MCI as a disconnection syndrome, at least in α and β frequency bands. This inconsistency on the results may be due to the heterogeneity of MCI, different neuroimaging techniques, different coupling measures, or a combination of these factors. Nonetheless, it has been demonstrated that the β band may have a special significance in AD, especially in the early stages. Our results showed that EEG activity in MCI patients is characterized by an overall similarity decrease in β_1_ band. Clinically, this reduced coupling may be due to the structural brain changes suffered by patients with MCI: decreased hippocampal volume, atrophy of the medial temporal lobe, or loss of gray matter volume (Karas et al., 2004).

Our findings suggest that EEG signals from different channels are more dissimilar among them in healthy people and they become gradually more similar as dementia progresses. It should be noticed the importance of β as the frequency band where the early changes in prodromal stages of AD are highlighted. Then, as the disease progresses, the abnormal coupling patterns also appear in low frequency bands, mainly at θ band in the current study, but also at α in previous ones (Koenig et al., 2005; Babiloni et al., 2006). These changes reflected the well-known disconnection syndrome and could be associated with both alterations in information processing at the cerebral cortex and to the disturbed synaptic transmission (e.g., decreased levels of acetylcholine), since they are associated with the complex dynamical processing within the brain neural networks (Baraniuk et al., 2001; Jeong et al., 2001).

### 5.3. Discrimination of MCI and AD patients from HC subjects

In order to evaluate the discrimination ability of *Cross-SampEn*, three different models (QDA, SVM, and DT) with two optimal set of features were used depending on the classification task (HC vs. MCI or HC vs. AD). Our results showed that the highest classification accuracy for HC vs. AD comparison was obtained using SVM and DT (*Acc* = 82.35%, *Se* = 70.59%, and *Sp* = 94.12%). Also SVM and DT obtained the highest accuracy for the HC vs. MCI classification problem (*Acc* = 73.53%, *Se* = 52.95%, and *Sp* = 82.35%). In our previous study (Ruiz-Gómez et al., 2018), where same training and test sets were used, we obtained precision values of 78.43 and 76.47% for HC vs. All and AD vs. All comparisons using a multi-layer perceptron artificial neural network, respectively. Our accuracy results using cross-entropy metrics are slightly better, but it should be noted that we are using different models for each binary classification instead of one multiclass model as in our previous work. Other previous studies achieved similar precision values, between 76.2 and 87.5% for AD vs. HC, and between 66.7 and 79.2% for MCI vs. HC (Huang et al., 2000; Poza et al., 2014, 2017; McBride et al., 2015). These results should be cautiously interpreted due to the use of different databases, usually with small sample sizes.

### 5.4. Limitations and future research lines

Despite the promising usefulness of *Cross-SampEn* as a measure to characterize brain dynamics in AD and its prodromal form, several limitations need to be addressed. Although we had a quite large database composed of 111 subjects, we divided our database to determine the optimal cross-entropy based metric and its configuration parameters that fit better to the characterization of coupling patterns in MCI and AD and to train and validate the models. It would also be possible to determine the optimal metric and its configuration with synthetic EEG signals generated from surrogate data with known dependencies or other models, as Kuramoto models (Acebrón et al., 2005). Moreover, it would be useful to conduct a longitudinal study of MCI subjects to gain a deeper understanding on the complex neural changes provoked by cognitive impairment; this would allow us to classify those with stable MCI and those who progress to AD. Finally, features derived from only one metric (*Cross-SampEn*) have been applied in this study to discriminate MCI and AD patients from HC subjects. It is noteworthy that in the case we would like to improve the classification ability of the models, features extracted from several other coupling metrics, like *SL, GFS*, phase-lag index (*PLI*) or directed transfer function (*DTF*), might also provide complementary information about neuronal alterations in this disorder that would improve the models.

## 6. Conclusions

This study provides original insights into the characterization of spontaneous neural activity in AD and MCI. *Cross-SampEn* has proven to be useful to gain a deeper understanding on the complex neural substrates underlying cognitive impairment preceding AD. Our results suggest that MCI and AD are associated with an overall similarity decrease between different brain regions, mainly at β_1_ frequency band. Furthermore, optimal FCBF-derived sets of these abnormalities have proved their usefulness to discriminate MCI and AD patients from controls, reaching relatively high accuracy values. These results highlight the usefulness of cross-entropy metrics in order to further understand the underlying brain dynamics in MCI and AD.

## Author contributions

SR-G processed the signals, analyzed the data, and wrote the manuscript. CG, MM-Z, and RH designed the study and interpreted the results. JP interpreted the results. MT-A and MC took part in the diagnosis of subjects and the collection of data. All authors have read and approved the final manuscript.

### Conflict of interest statement

The authors declare that the research was conducted in the absence of any commercial or financial relationships that could be construed as a potential conflict of interest.
